# Histological Stratification of Thick and Thin Plaque Psoriasis Explores Molecular Phenotypes with Clinical Implications

**DOI:** 10.1371/journal.pone.0132454

**Published:** 2015-07-15

**Authors:** Jaehwan Kim, Pranay Nadella, Dong Joo Kim, Carrie Brodmerkel, Joel Correa da Rosa, James G. Krueger, Mayte Suárez-Fariñas

**Affiliations:** 1 Laboratory for Investigative Dermatology, The Rockefeller University, New York, New York, United States of America; 2 Harvard University, Cambridge, Massachusetts, United States of America; 3 School of Medicine, Stony Brook University, Stony Brook, New York, United States of America; 4 Immunology & Biomarkers, Janssen Research & Development, Radnor, Pennsylvania, United States of America; 5 The Center for Clinical and Translational Science, The Rockefeller University, New York, New York, United States of America; 6 Icahn School of Medicine at Mount Sinai, New York, New York, United States of America; CNRS-University of Toulouse, FRANCE

## Abstract

Psoriasis, which presents as red, scaly patches on the body, is a common, autoimmune skin disease that affects 2 to 3 percent of the world population. To leverage recent molecular findings into the personalized treatment of psoriasis, we need a strategy that integrates clinical stratification with molecular phenotyping. In this study, we sought to stratify psoriasis patients by histological measurements of epidermal thickness, and to compare their molecular characterizations by gene expression, serum cytokines, and response to biologics. We obtained histological measures of epidermal thickness in a cohort of 609 psoriasis patients, and identified a mixture of two subpopulations—thick and thin plaque psoriasis—from which they were derived. This stratification was verified in a subcohort of 65 patients from a previously published study with significant differences in inflammatory cell infiltrates in the psoriatic skin. Thick and thin plaque psoriasis shared 84.8% of the meta-analysis-derived psoriasis transcriptome, but a stronger dysregulation of the meta-analysis-derived psoriasis transcriptome was seen in thick plaque psoriasis on microarray. RT-PCR revealed that gene expression in thick and thin plaque psoriasis was different not only within psoriatic lesional skin but also in peripheral non-lesional skin. Additionally, differences in circulating cytokines and their changes in response to biologic treatments were found between the two subgroups. All together, we were able to integrate histological stratification with molecular phenotyping as a way of exploring clinical phenotypes with different expression levels of the psoriasis transcriptome and circulating cytokines.

## Introduction

Psoriasis is a common skin disease affecting 2 to 3 percent of the world population. It begins as red, scaly patches on the scalp, elbows, and knees that, if it progresses to severe disease, is associated with systemic inflammation and comorbidities, such as psoriatic arthritis, cardiovascular disease, diabetes, and depression [1–7]. Despite having a complex, multifactorial autoimmune disease etiology, our understanding of psoriasis has been rapidly expanding along with the availability of high throughput technologies for comprehensive molecular characterization [8, 9]. Through genomic analysis, important pathogenic molecules have been identified, and a broad spectrum of anti-psoriatic agents has been developed and has been proven to be highly effective [10–13].

However, there is still a gap between the molecular findings in the laboratory and the personalized treatment of psoriasis in real-world practice. A more complete understanding of these molecular characteristics, and the extent to which they differ between individual psoriasis patients, would provide valuable insights to their treatment. Clinical observation alone would support the idea of a spectrum of clinical disease phenotypes that includes small and large plaque psoriasis [14], as well as notable variants like guttate and plaque forms of psoriasis. This raises the hypothesis that there are various clinical forms of psoriasis each with their own molecular profiles.

Epidermal thickness is assessed indirectly in the Psoriasis Area Severity Index (PASI) as plaque elevation, and independently identifies two morphologic subpopulations (“thick” and “thin”) [14]. Patients with thick plaques tend to associate with higher BMI and psoriatic arthritis, while patients with thin plaques tend to associate with guttate psoriasis and skin cancer [14]. Because measuring the epidermal thickness on an image of skin biopsy tissue provides the most accurate assessment of skin thickness, we investigated whether or not histological measurements of epidermal thickness can be used to stratify psoriasis patients into subgroups with different expression levels of the psoriasis transcriptome and different levels of circulating cytokines in response to biologic treatments. To explore the existence of subpopulations using the measurement of epidermal thickness, we first examined the distribution of histologically measured epidermal thickness of 609 psoriasis patients from the de-identified data accumulated at our tissue bank (S1 Table). As the analysis revealed two underlying distributions of epidermal thickness differences between lesional and non-lesional skin, we compared the number of inflammatory cells, expression profiles, circulating cytokines, and treatment responses to biologics between subpopulations from a subset of patients where immunohistochemical and microarray data was available [15, 16]. Since we analyzed data from a single study on a single platform, we could minimize batch effects that may be seen across different studies. To our knowledge, this is the first description correlating epidermal thickness, psoriasis transcriptome, circulating cytokines, and clinical responses to biologics.

## Materials and Methods

### The experimental data

The tissue bank in the Laboratory for Investigative Dermatology, Rockefeller University, provided de-identified epidermal thickness data of 609 patients with moderate-to-severe psoriasis (http://lab.rockefeller.edu/krueger/) (S1 Table). The data from the tissue bank had been accumulated from multiple clinical trials approved by the Rockefeller University Institutional Review Board.

The gene expression data from skin biopsy samples of moderate-to-severe psoriasis patients was obtained from the NIH’s GEO (Gene Expression Omnibus) repository (GSE30999). The skin biopsy samples were collected from histologically confirmed psoriasis patients who were enrolled into an IRB-approved Phase 3, multicenter, randomized trial protocol (ACCEPT trial)[15]. The platform of expression profiling was Affymetrix Human Genome U133 Plus 2.0 Array. The raw Affymetrix data (CEL files) were downloaded from GEO repository and expression values were obtained using GCRMA algorithm [17], while normalization across samples was carried out using quantile normalization. As the first step of data filtering, only those probe sets with at least 1 sample with expression values larger than 3 and standard deviation/SD >0.1 were kept for further analyses. RT-PCR data was generated using Applied Biosystems (Foster City, CA) Taqman gene expression assays and Taqman low-density array cards. The microarray and RT-PCR data, and relevant clinical information were published and publicly accessible [16]. Serum cytokine data was obtained from a subcohort of the microarray and RT-PCR studies [16], as well as from repository data of the tissue bank in the Laboratory for Investigative Dermatology, Rockefeller University.

### Epidermal thickness and inflammatory cell infiltrates measurement

Epidermal thickness was measured from frozen sections of psoriatic lesional and non-lesional skin biopsies. Tissues were stained with hematoxylin (Fisher Scientific, Pittsburgh, PA) and eosin (Shandon, Pittsburgh, PA), and images were taken under microscope with 10× magnification. Epidermal thickness was then measured with the ImageJ program [18]. As psoriatic lesional skin should presumably be thicker than non-lesional skin of the same patient, patient data whose lesional skin was thinner than non-lesional skin was excluded for further analysis. Inflammatory cell infiltrates were counted from frozen sections of psoriatic lesional skin biopsies. The immunostained cells in the dermis of skin were counted from a representative image captured under microscope with 10× magnification with the ImageJ program [18].

### Statistical Analysis

Statistical methods to analyze histologically measured epidermal thicknesses were primarily based on the hypothesis of the presence of *k* morphologic subpopulations. This hypothesis was translated in statistical terms to modeling epidermal thickness by a finite mixture of *k* Gaussian distributions, *k* being an unknown parameter. The strategy can be seen as a model-based clustering approach to allocate individuals in their morphologic subpopulations and simultaneously estimate the parameters of their underlying Gaussian distributions. Under this framework, we found inferential evidence of two existing subpopulations in 609 psoriasis patients, labeled hereafter as Thin/Thick Psoriasis, centered at different magnitudes of epidermal thickness with unequal variances (Table 1). Conditional on these findings, extensive data analysis was carried in a subcohort of 65 psoriasis patients to gather evidence that gene expression, pathway activity, and inflammatory cell counts differed between the two identified subpopulations. Additionally, linear mixed-effects models were fitted to test and estimate the effects of interaction between thickness and tissue type (lesional/non-lesional).

**Table 1:** Histologically measured epidermal thickness of 609 moderate-to-severe psoriasis patients.

		Thin Psoriasis	Thick Psoriasis	Total
Number of samples		285	324	609
Lesional skin (μm)	Mean	255.5	450.6	359.3
	SEM^1^	59.6	93.4	5.1
Non-lesional skin (μm)	Mean	90.0	89.8	89.9
	SEM^1^	1.6	1.3	1.0

^1^SEM: Standard Error of the Mean

“Thick” and “thin” plaque psoriasis were defined above and below 250 μm of the difference between lesional and non-lesional epidermal thickness.

#### Mixture of Distributions

In order to identify the number of morphologic subpopulations (components in a final Gaussian mixture model) and estimate their parameters, R package “mixtools” was used. A robust likelihood ratio statistic obtained from 1000 bootstrap realizations was used to test for the smallest number k of components compatible with the data. Sequentially testing the null hypothesis of a k-component fit versus the alternative hypothesis of a (k+1)-component fit, our iterations stopped when the null hypothesis of *k* = 2 was not rejected in 609 psoriasis patients. The parameters of the mixture model were estimated using the *Expectation Maximization (EM) Algorithm* [19] as implemented in R package normalmixEM. Parameter estimates for the mixed Gaussian distributions keep the same magnitude when the EM algorithm is applied to the subcohort of 65 psoriasis patients where complete gene array profile is available.

#### Analysis of Arrays, RT-PCR, and circulating cytokines

For microarray and RT-PCR data, log_2_-transformed expression values were modeled using linear mixed-effects models with skin thickness (thick/thin) and tissue type (lesional/non-lesional) as fixed effects and a random intercept for each subject. After fitting the interaction model, comparisons of interest were assessed using linear contrasts via restricted log-likelihood maximization (REML), under the general framework of *limma* package. *P*-values from moderated (paired) t-test were adjusted for multiple hypotheses across genes using Benjamini-Hochberg procedure.

#### Pathway Analysis

The activity of entire signaling pathways for each sample was quantified by using a per-patient GSEA (Gene Set Enrichment Analysis)-like method. GSVA is an unsupervised sample-wise enrichment method described in [20]. Enrichment scores were obtained by setting the parameter z-score in gsva function available in *R* package. The formulation for scores evaluation is described in [21]. The authors proposed the linear combination of normalized expression with weights in the combination being defined as $1/\sqrt{k}$ for *k* being the number of genes in the pathway. This methodology was applied to obtain pathway enrichment scores in both microarray and RT-PCR analyses. These enrichment scores were used as inputs for the same linear mixed model framework, described previously for gene expression, in order to evaluate significant differences in thick versus thin plaques.

#### Correlation Analysis of Epidermal Thickness versus Markers for inflammation

Association between histologically measured epidermal thickness and counts for different inflammatory cells was explored through a multiple linear regression model that included epidermal thickness as the dependent variable and markers for inflammation as predictors. The selection of a subset of markers significantly correlated with epidermal thickness was carried by forward stepwise selection as implemented in IBM SPSS statistics Version 22. Partial correlation coefficients measured by beta regression coefficients were used to measure degree of association between a specific marker and the epidermal thickness.

#### Treatment response analysis

The change in PASI was modeled using mixed effect models with time and treatment group as fixed effects and random intercept for each patient. Estimates for the change in treatment were obtained using least squares means and represented as percent of improvement.

Most of the statistical analysis was carried out in the R language version 2.12 (www.r-project.org), and packages were from the Bioconductor project (www.bioconductor.org). IBM SPSS Statistics Version 22 was used for forward stepwise linear regression modeling.

## Results

### “Thick” and “thin” plaque psoriasis identified using an *expectation maximization algorithm*

**Fig 1:**
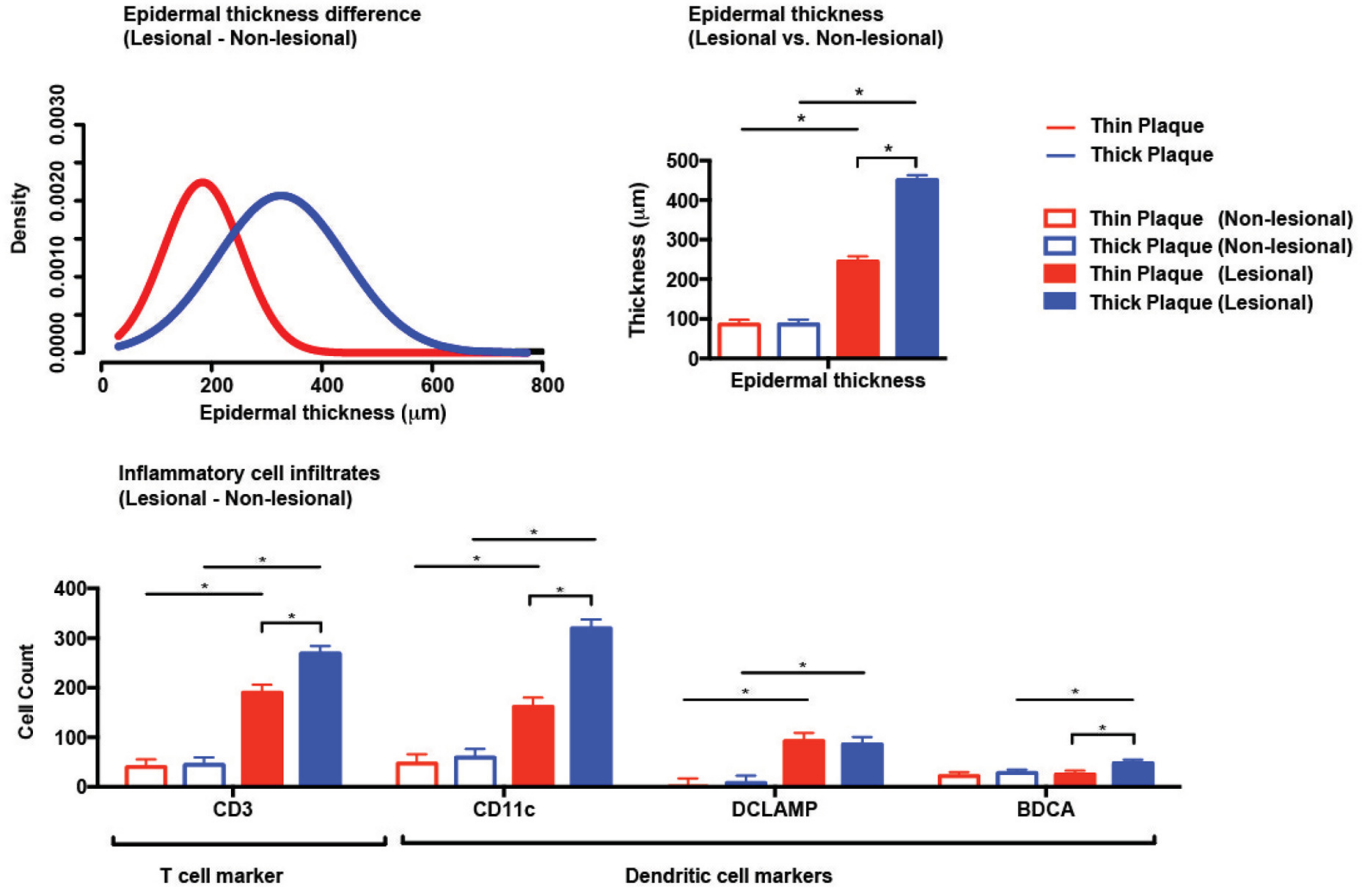
**(A) Difference in epidermal thickness between lesional and non-lesional skin.** Expectation maximization algorithm identifies two underlying distributions of the epidermal thickness difference (thick and thin plaque psoriasis). **(B) Epidermal thickness of thick and thin plaque psoriasis.** Epidermal thickness is significantly different between thick and thin plaque psoriasis in the lesional skin (n = 609 lesional and non-lesional matched biopsies, *p* <0.05, ANOVA, Tukey’s HSD test). **(C) Inflammatory cell infiltrates in the dermis of thick and thin plaque psoriasis.** CD3 ^+^ T cells and CD11c ^+^ dendritic cells are significantly different between thick and thin plaque psoriasis in the lesional skin (n = 65, *p* <0.05). Bars represent means; error bars represent standard error of the mean (SEM).

The differences in histologically measured epidermal thickness were calculated from the lesional and matched non-lesional biopsies of 609 psoriasis patients (S1 Table), and we evaluated whether the distribution arose from multiple populations by fitting a mixture model through an expectation maximization algorithm (see Materials and Methods) [22]. Using the likelihood ratio statistic, we tested the null hypothesis of all values from the same distribution versus the alternative hypothesis of multiple subpopulations in the distribution. The test result rejected the null hypothesis (*p* = 0.007) and identified two underlying distributions of the epidermal thickness difference (Fig 1A). As the two distribution curves intersected at 250 μm, “thick” and “thin” plaque psoriasis were defined above and below 250 μm as the difference between lesional and non-lesional epidermal thickness (Table 1). There was a significant difference in epidermal thickness of psoriatic lesions between thick and thin plaque psoriasis, while no difference was observed between thick and thin plaque psoriasis in non-lesional skin biopsies (Fig 1B).

### Immunohistological validation of “thick” and “thin” plaque psoriasis

We next explored the biological implications of the epidermal thickness-based stratification in a subcohort of 65 moderate-to-severe plaque psoriasis patients [16]. The analysis of demographic information confirmed that phenotyping of thick and thin plaque psoriasis was not associated with differences in gender, age, BMI or duration of psoriasis (Table 2). Importantly, PASI or Body Surface Area (BSA) was not different between thick versus thin plaque psoriasis. Although PASI assesses the induration of psoriatic skin, the overall score did not reflect differences in histologically measured epidermal thickness.

**Table 2:** Demographic and clinical characteristics of thick and thin plaque psoriasis.

		Plaque Psoriasis		
Characteristics		Thin (n = 31)	Thick (n = 34)	*p*
Sex, No (%)	Female	6 (19.4)	7 (20.6)	.667
	Male	25 (80.6)	27 (79.4)	
Age, y		43	46	.473
Duration of psoriasis (yr)		17.5	17.9	.896
BMI		32.3	33.8	.421
PASI^1^ (0~72)		22.3	20.2	.456
BSA^2^ (0~100%)		33.0	26.8	.229

^1^PASI: Psoriasis Area and Severity Index

^2^BSA: Body Surface Area

To verify whether or not our stratification of psoriasis patients into subgroups drew meaningful differences in psoriasis immunopathogenesis, the numbers of T cells and dendritic cells in the dermis of the skin were compared between thick and thin plaque psoriasis. The numbers of CD3^+^ T cells, CD11c^+^ myeloid dendritic cells, and BDCA-1^+^ resident dendritic cells were significantly different in the lesional skin between subgroups, while no differences were observed in non-lesional skin (Fig 1C). Additionally, dendritic cells and T cells numbers were significantly correlated with epidermal thickness in the linear regression model (correlation coefficient: CD11c^+^ dendritic cells = 0.836, CD3^+^ T cells = 0.252; S1 Fig).

### Thick and thin plaque psoriasis share the psoriasis transcriptome but the expression levels are different

To understand thick versus thin plaque psoriasis phenotypes at the genomic level, we compared the gene expression profiles of lesional and non-lesional psoriasis across the two groups, and tried to elucidate: 1) different gene sets, with the expression of different gene sets explaining the difference of phenotypes, and 2) identical gene sets, with different expression levels of the identical gene sets determining the difference of phenotypes.

**Fig 2:**
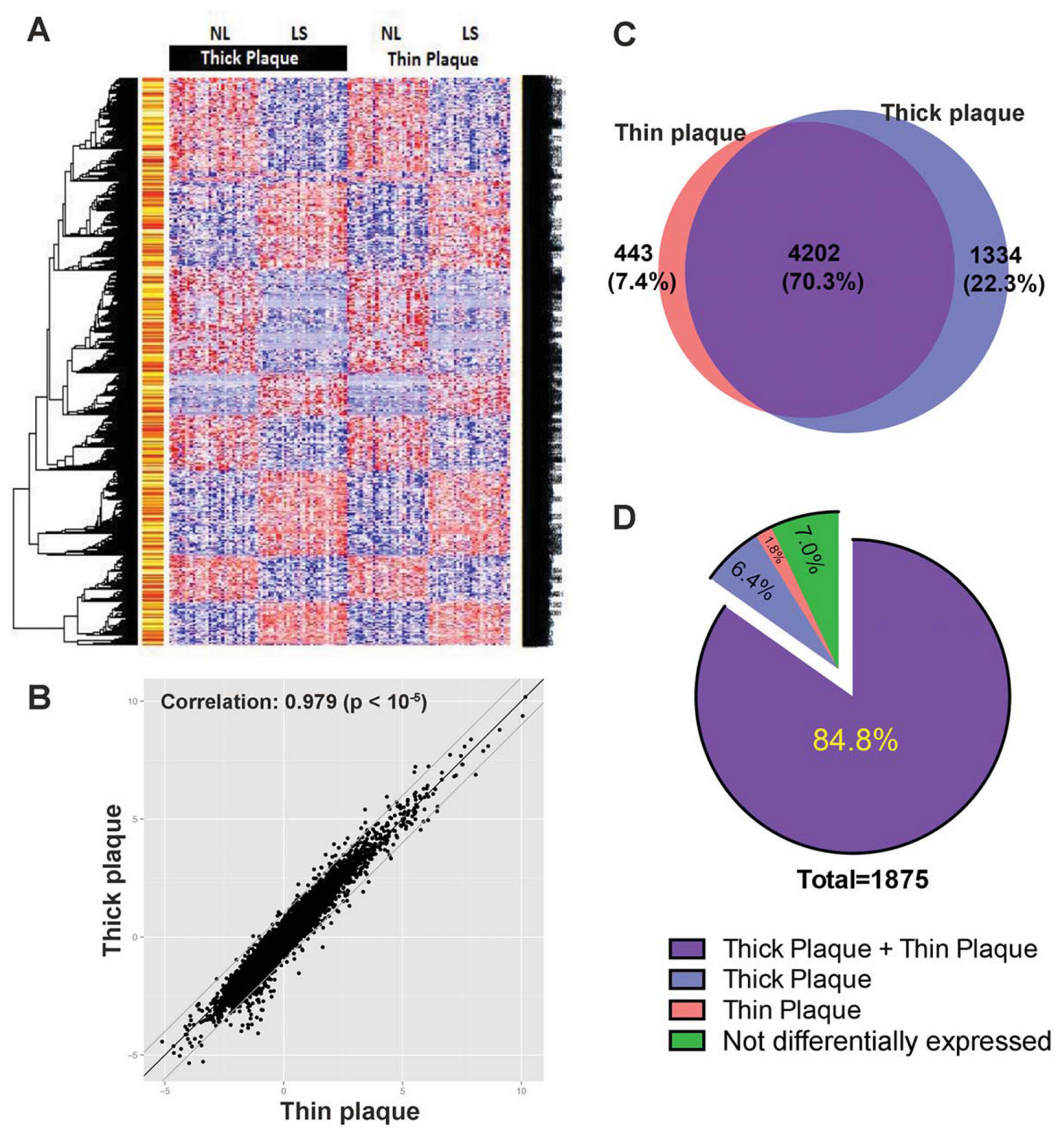
Comparison of differentially expressed genes (DEGs) between thick and thin plaque psoriasis. (A) Heatmap that displays the 12,001 differentially expressed probesets (FCH >2.0, *p* <0.05, and FDR < 0.05). The distance matrix was built based on Euclidean distances and the McQuitty algorithm was used for clustering. (B) Scatter plot that displays the high correlation of DEGs between thick and thin plaque psoriasis (log_2_ fold change). (C) Area-proportional Venn diagram comparing DEGs between thick and thin plaque psoriasis (>2-fold change and <0.05 false discovery rate). (D) Differentially expressed genes among the meta-analysis derived transcriptome genes (MAD3, *Tian et al*., 2012).

We examined gene expression profiles (GEO series GSE30999) in a subcohort of 65 moderate-to-severe plaque psoriasis patients who had histological confirmation of psoriasis [16], and DEGs between lesional versus uninvolved or non-lesional skin were compared at the classical cutoff of FCH > 2.0, *p* < 0.05, and FDR < 0.05 (Fig 2A). Fold changes of DEGs were highly correlated between thick and thin plaque psoriasis (correlation coefficient = 0.979; Fig 2B), and 70.3% of them were overlapped (Fig 2C). For confirmation, we compared a meta-analysis-derived (MAD3) transcriptome as a standard reference list of psoriasis transcriptomes [23] and found that 84.8% of the MAD3 transcriptome was shared by thick and thin plaque psoriasis (Fig 2D).

**Fig 3:**
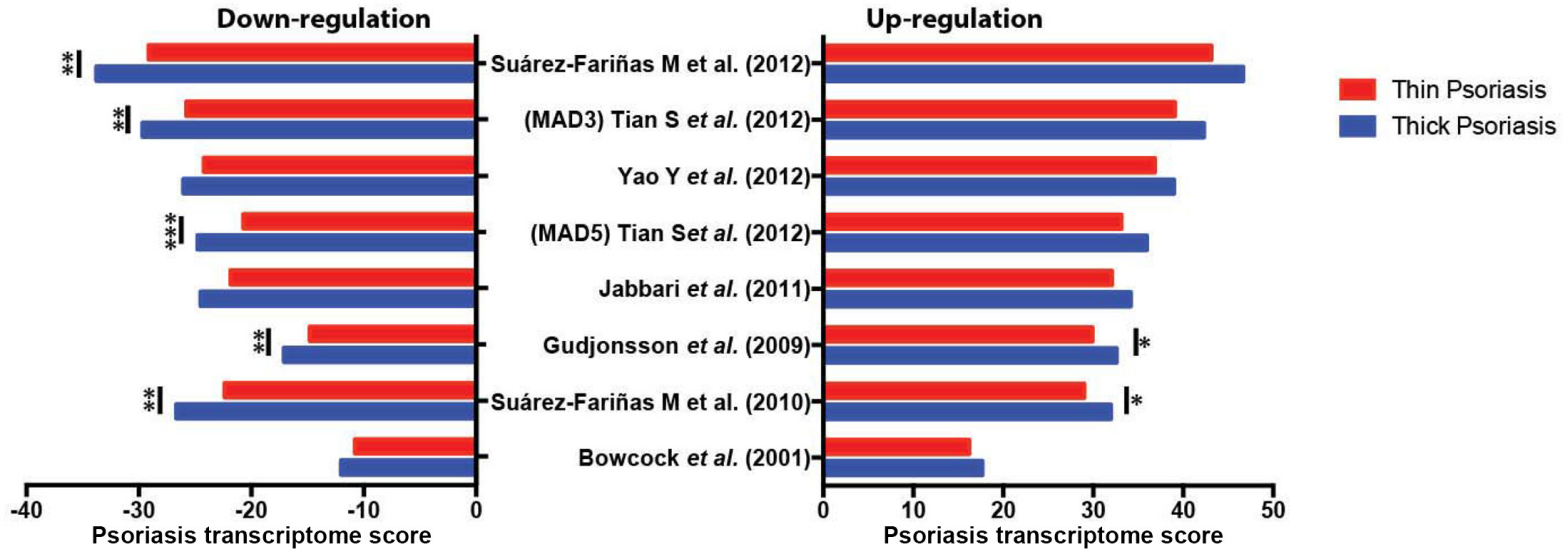
Comparison of psoriasis transcriptome scores between thick and thin plaque psoriasis. Based on psoriasis transcriptome references, psoriasis transcriptome scores are calculated by the combined z-score gene set variation analysis (GSVA) method. The absolute values of psoriasis transcriptome scores are consistently higher in thick plaque psoriasis compared to those of thin plaque psoriasis (*p <0.*1*, **p <0.*05*, ***p <0.*01)*.

To investigate different expression levels of the psoriasis transcriptome between thick and thin plaque psoriasis skin, we performed gene set variation analysis (GSVA) via the combined z-score method [21]. Using the established psoriasis transcriptome references [16, 23–28], psoriasis transcriptome scores in each lesional or non-lesional biopsy sample were computed from the observed gene expression levels. As shown in Fig 3, the absolute values of all the psoriasis transcriptome scores in thick plaque psoriasis were consistently higher than those in thin plaque psoriasis. It is noteworthy that the pattern is driven more by the down-regulation scores (Fig 3, left), rather than the up-regulation scores (Fig 3, right).

In addition, we investigated if the expression levels of psoriasis genes included in the MAD3 transcriptome were different between lesional versus non-lesional skin, or if the magnitude of dysregulation was different between thick and thin plaque psoriasis. Important genes in the MAD3 transcriptome involved in keratinocyte proliferation (Ki-67), keratinocyte adhesion (cadherin 26 and desmocollin 2), T_H_17-regulated cytokines (IL-1B, IL-36A, CXCL1, CXCL2, and CXCL8), T_H_1-regulated cytokines (CXCL11), anti-inflammatory cytokine (IL-37), lipid metabolism (LEP, LPL, APOBEC3A, APOBEC3A, CH25H, and APOD), cardiovascular disease (RBP4), and angiogenesis (ANG) were dysregulated between lesional versus non-lesional skin more strongly in thick plaque psoriasis compared to thin plaque psoriasis (Table 3). Especially IL-36A was noted, as recent studies have found that IL-36 cytokines, that belong to IL-1 family, have significant role in regulation of psoriasis and psoriasis activity [29–31]. Together, the data support the notion that the differences between thick versus thin plaque psoriasis phenotypes are determined by different degrees of dysregulation of the largely identical psoriasis transcriptome.

**Table 3:** Comparing differentially expressed genes (DEGs) included in the meta-analysis-derived (MAD3) transcriptome between thick and thin plaque psoriasis.

CATEGORY	SYMBOL	GENENAME	LS vs NL		Thick vs Thin	
			Thin	Thick	NL	LS
Keratinocyte hyperproliferation	MKI67	marker of proliferation Ki-67	4.3***	6.8***	-1.0	1.6**
Keratinocyte adhesion	CDH26	cadherin 26	4.3***	15.4***	1.0	3.6***
	DSC2	desmocollin 2	13.5***	23.8***	1.4	2.5***
	PKP1	plakophilin 1	6.5***	18.3***	-2.3*	1.2
Cell adhesion molecules	CDHR1	cadherin-related family member 1	-3.7***	-4.6***	-1.4	-1.7**
T_H_17-regulated cytokines	IL1B	interleukin 1, beta	4.6***	9.1***	1.1	2.2**
	IL36A	interleukin 36, alpha	3.0***	7.7***	1.0	2.6***
	CXCL1	chemokine (C-X-C motif) ligand 1	45.1***	127.5***	1.1	3.1***
	CXCL2	chemokine (C-X-C motif) ligand 2	13.3***	18.2***	1.7	2.3**
	CXCL8	chemokine (C-X-C motif) ligand 8	68.2***	150.7***	1.0	2.3*
T_H_1-regulated cytokines	CXCL11	chemokine (C-X-C motif) ligand 11	1.5***	3.0***	-1.0	2.0**
Anti-inflammatory	IL37	interleukin 37	-3.5***	-6.8***	-1.6	-3.1**
Lipid metabolism	LEP	Leptin	-2.8***	-14.5***	4.1**	-1.3
	LPL	lipoprotein lipase	-2.2***	-5.4***	2.2*	-1.1
	APOBEC3A	apolipoprotein B mRNA editing enzyme, catalytic polypeptide-like 3A	4.1***	10.6***	-1.0	2.6**
	CH25H	cholesterol 25-hydroxylase	4.1***	6.2***	1.2	1.8**
	APOD	apolipoprotein D	-1.7***	-2.0***	-1.1	-1.3**
Cardiovascular disease	RBP4	retinol binding protein 4, plasma	-2.1***	-16.9***	4.4**	-1.8
Angiogenesis	ANG	angiogenin, ribonuclease, RNase A family, 5	-3.2***	-5.7***	-1.3	-2.2***

LS: Lesional skin

NL: Non-lesional skin

***FDR <0.05

**FDR <0.1

*FDR <0.2

### Gene expression of thick and thin plaque psoriasis is different not only within psoriatic lesional skin but also in peripheral non-lesional skin

**Fig 4:**
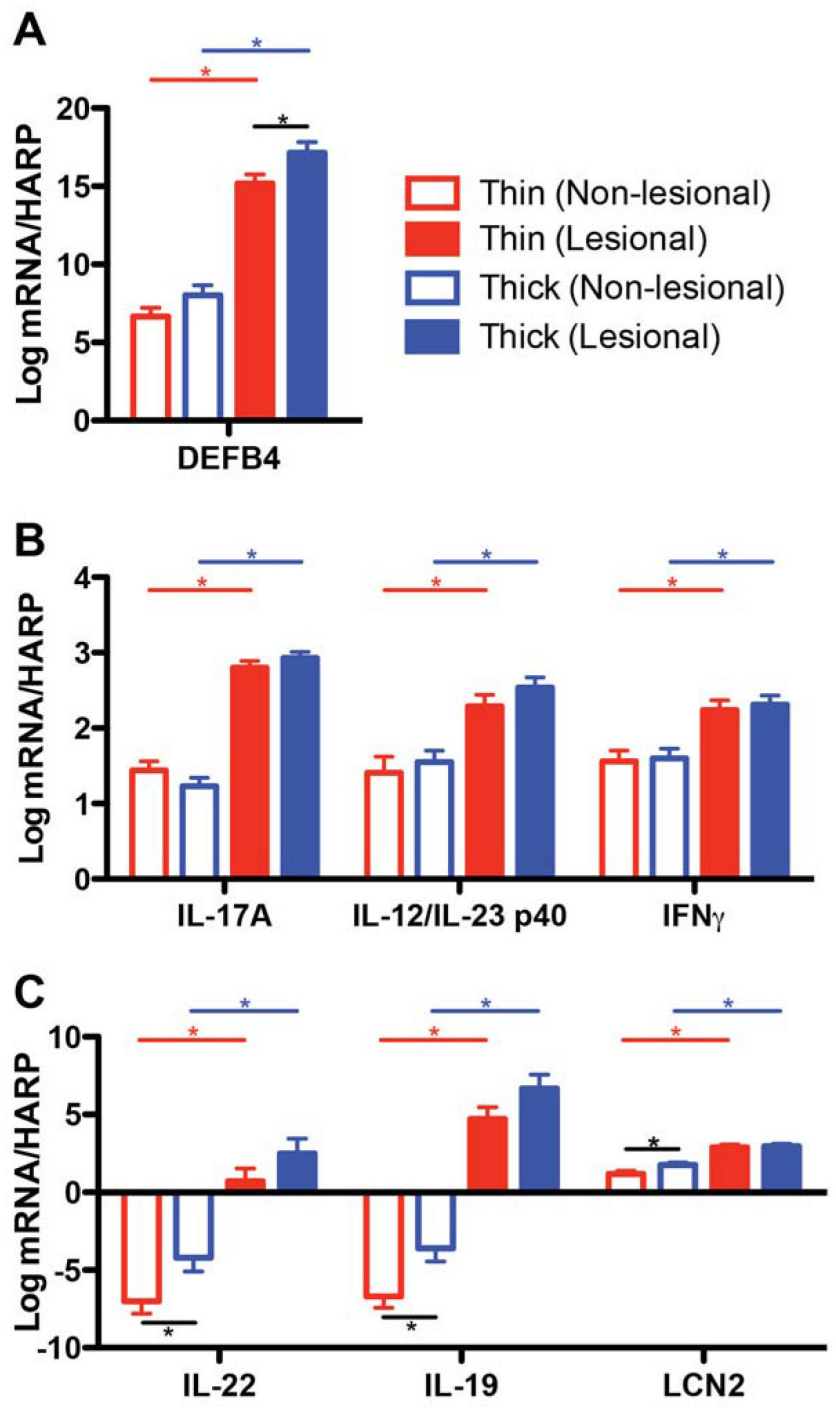
Comparison of psoriatic gene expression normalized to the house-keeping gene, human acidic ribosomal protein (hARP). (A) Gene expression is significantly different only within lesional skin. (B) Gene expression is not significantly different within both lesional and non-lesional skin. (C) Gene expression is significantly different only within non-lesional skin (n = 16 for thick plaque psoriasis, n = 20 for thin plaque psoriasis; *p < 0.05; bars represent means; error bars represent SEM).

To better understand the different degrees of dysregulation of the psoriasis transcriptome between thick and thin plaque psoriasis, the expression of 70 key genes of the psoriasis transcriptome was investigated by RT-PCR in a subcohort of patients (16 thick plaque psoriasis and 20 thin plaque psoriasis). Among them, the expression of 23 genes was dysregulated between lesional versus non-lesional skin in both thick and thin plaque psoriasis (FDR <0.05). Within the expression data, we stratified three different dysgregulation patterns of key cytokines as described in Fig 4.

DEFB4, a T_H_17-regulated antimicrobial peptide produced by keratinocytes, followed a trend towards increased lesional versus non-lesional dysregulation in thick plaque psoriasis compared to thin plaque psoriasis (0.05< *p* <0.10). The expression level of DEFB4 in thick plaque psoriasis was higher than the expression level in thin plaque psoriasis within lesional skin, while no difference was seen within non-lesional skin (Fig 4A). On the other hand, IL-17A, IL-12/IL-23 p40, and IFN-γ, key molecules implicated in psoriasis pathogenesis [32], revealed no differences between thick and thin plaque psoriasis within both lesional and non-lesional skin (Fig 4B). Interestingly, the expression levels of downstream genes in IL-17 signaling pathways, IL-19 and LCN2, as well as IL-22, were higher in thick plaque psoriasis compared to thin plaque psoriasis within non-lesional skin, while there were no significant differences within lesional skin (Fig 4C). Since IL-17 levels were similar but its downstream pathway product levels were different within non-lesional skin of thick and thin plaque psoriasis, we hypothesized that systemic inflammatory differences may exist between the subtypes and further analyzed the expression of circulating cytokines and other inflammatory factors in blood.

### Different levels of circulating cytokines and their changes in response to biologics between thick and thin plaque psoriasis

**Fig 5:**
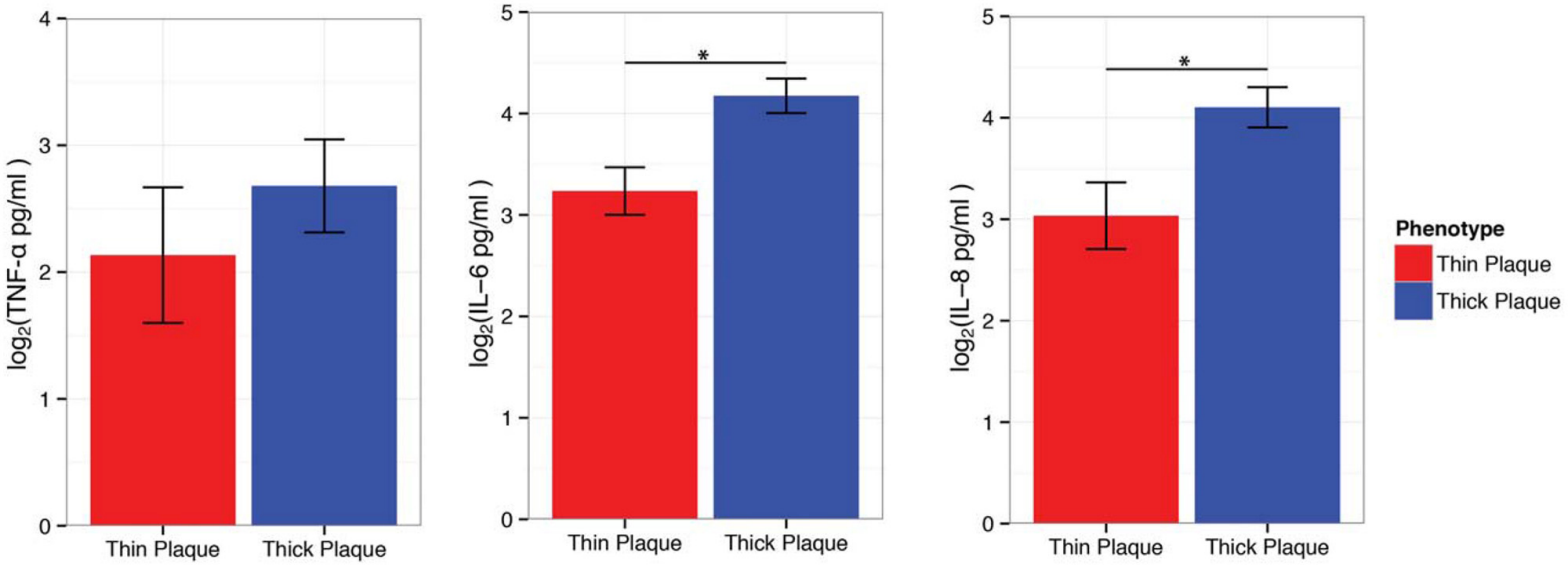
Comparison of serum TNF-α, IL-6, and IL-8 between thick and thin plaque psoriasis. Serum levels of TNF-α were not significantly different (*p* = 0.11), but its downstream cytokines, IL-6 and IL-8, were significantly higher in thick plaque psoriasis compared to thin plaque psoriasis (*p* = 0.0035 for IL-6, *p* = 0.0036 for IL-8) for a mixed effect model with a random intercept for the cohort (n = 19 for thick plaque psoriasis, n = 7 for thin plaque psoriasis; bars represent means; error bars represent SEM).

Within a subset of patients with available serum cytokine measures (n = 26), we stratified patients into thick plaque psoriasis (n = 19) and thin plaque psoriasis (n = 7), and investigated different levels of circulating cytokines [16]. Serum levels of TNF-α showed a trend for higher expression in thick plaque psoriasis compared to thin plaque psoriasis (*p* = 0.11), and its downstream cytokines, IL-6 and IL-8, were significantly higher in thick plaque psoriasis in comparison to thin plaque psoriasis (*p* = 0.0035 for IL-6, *p* = 0.0036 for IL-8; Fig 5). Since serum TNF-α is increased in active psoriasis and correlates with disease severity [33, 34], higher levels of its downstream cytokines, IL-6 and IL-8, in thick plaque psoriasis likely reflects more biologic activity of TNF-α in thick plaque psoriasis.

**Fig 6:**
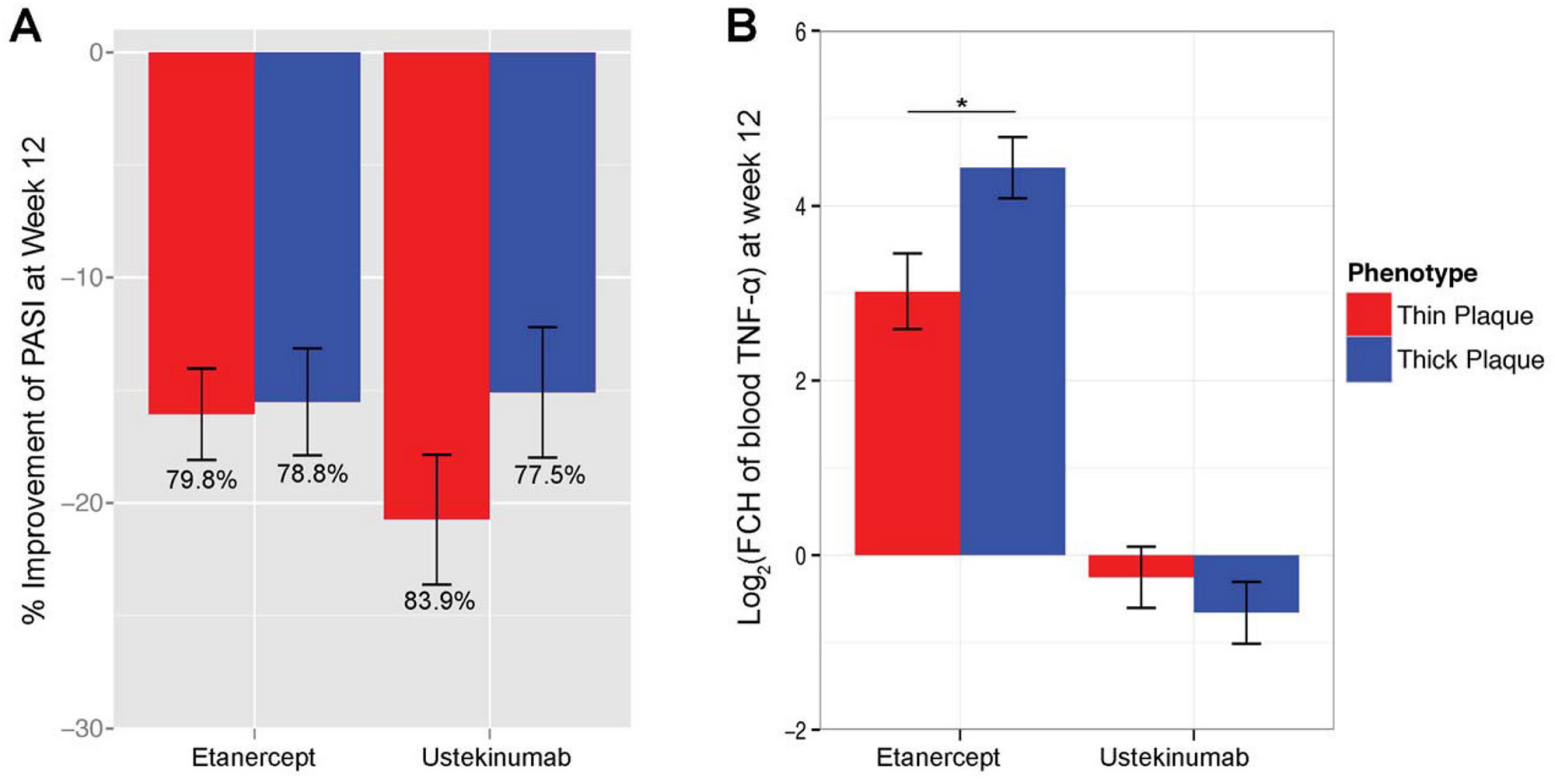
Comparison of treatment response between thick and thin plaque psoriasis. (A) Percent improvement in Psoriasis Area Severity Index (PASI) is compared between Etanercept (12 with thick plaque psoriasis and 15 with thin plaque psoriasis) and Ustekinumab 90 mg treatment (12 with thick plaque psoriasis and with 12 thin plaque psoriasis). (B) Serum TNF-α increased more in thick plaque psoriasis after 12 weeks of Etanercept treatment (*p* = 0.04). In contrast, it decreased with Ustekinumab treatment and there was no significant difference between thick and thin plaque psoriasis (*p* >0.05). Bars represent means; error bars represent SEM.

We next compared changes in the cytokine levels in response to biologics. When we compared clinical response by percent improvement in PASI, there was no significant difference between thick and thin plaque psoriasis after 12 weeks of treatment with Etanercept or Ustekinumab (*p* >0.05, Fig 6A). In serum, TNF-α levels decreased with Ustekinumab treatment and there was no significant difference between thick and thin plaque psoriasis (*p* >0.05, Fig 6B). The serum level of TNF-α after Ustekinumab treatment reflected a direct measure of final amount of TNF-α after the treatment, as Ustekinumab is a human immunoglobulin monoclonal antibody that binds with high affinity to the shared P40 subunit of human interleukins 12 and 23, inhibiting their binding to the interleukin 12Rβ1 receptor [35].

Paradoxically, TNF-α in serum increased after 12 weeks of Etanercept treatment and the increase was significantly higher in thick plaque psoriasis compared to thin plaque psoriasis (*p* = 0.04), reflecting more circulating TNF-α in thick plaque psoriasis (Fig 6B). Since Etanercept is a genetically engineered fusion protein that binds and inactivates TNF [36], the serum level of TNF-α after Etanercept treatment reflected total amount of TNF-α before the treatment in conjunction with the increased amount of TNF-α during the treatment as sequestered in circulating TNF-etanercept complexes.

## Discussion

Clinical phenotyping of thick and thin plaque psoriasis was first described in 2006 by Christensen *et al*. [14]. Based on clinical data collected prospectively from 500 psoriasis patients in the Utah Psoriasis Initiative, the study described differences between thick and thin plaque psoriasis in regards to gender, BMI, psoriatic arthritis, eczema, guttate psoriasis, and skin cancer. We drew inspiration from this study, but approached phenotyping in a different way. Instead of defining thick and thin plaque psoriasis based on the physician assessment score and patient-reported psoriasis plaque thickness at their worst, we utilized histologically measured epidermal thickness data and applied a statistical algorithm to define thick and thin plaque psoriasis. In contrast to the previous study, our stratification does not reflect clinical prognosis over time but demonstrates a status at a single time point. However, our method assures objective stratification of thick and thin plaque psoriasis that excludes the potential bias that can arise from subjective measurement of epidermal thickness by different physicians and patients. Christensen *et al*. [14] reported that thick plaque psoriasis was associated with male gender and increased BMI, but thick versus thin plaque psoriasis assignment was not associated with gender or BMI in our study (Table 2). This discrepancy in clinical observation is likely due to differences in our phenotyping approach.

We confirmed the validity of histological phenotyping thick and thin plaque psoriasis by significant differences of immunopathogenic cell numbers between groups. Then, the subgroups were compared to understand the correlation between epidermal thickness and genomic profiling. Rather than on-and-off activation or suppression of individual genes, gene expression levels of a similar transcriptome were increased or decreased in accordance with the changes of epidermal thickness. It is noteworthy that we profiled different patterns of gene expression between thick and thin plaque psoriasis not only within psoriatic lesional skin but also in non-lesional skin by RT-PCR. Keermann *et al*. [29]. recently compared transcriptional changes of psoriatic lesional skin, non-lesional skin, and normal skin from healthy controls, and showed that non-lesional skin of psoriasis patients is affected by psoriasis-like gene expression profiles. Our study also supports the notion that non-lesional skin of psoriasis patients is diseased skin, as some important psoriasis genes, such as IL-22, IL-19 and LCN2, were differentially expressed between thick and thin plaque psoriasis only within non-lesional skin (Fig 4C). The existence of a disease phenotype in non-lesional skin of thick plaque psoriasis may represent effects of circulating cytokines to generate the phenotype. Thick plaque psoriasis has greater amounts of mRNA in the skin and therefore more cytokines are produced in psoriatic plaques. The cytokines from the psoriatic plaque spread into the circulation, which was represented by high serum levels of TNF-α downstream cytokines, IL-6 and IL-8, in thick plaque psoriasis. As a result, circulating inflammatory cytokines may induce downstream genes in IL-17 signaling pathways in non-lesional skin to produce a disease phenotype.

We compared gene expression normalized to the house-keeping gene human acidic ribosomal protein (hARP). However, when we quantified the total amount of mRNA extracted from the skin biopsy, we found that the amount of mRNA in thick plaque psoriasis was 3-fold change higher than the amount of mRNA in thin plaque psoriasis, in both lesional and non-lesional skin (*p* = 0.02 and 0.005 for lesional and non-lesional skin, respectively). Thus, we posit that overall production of inflammatory cytokines in thick plaque lesions would be over 3 times higher in thick plaque psoriasis versus thin plaque psoriasis if the total mRNA amount were taken into account. Another limitation of this study is that multiple plaques per patient were not sampled. As only one biopsy was taken of a typical psoriasis lesion from each psoriasis patient, lesion-to-lesion variation that might be associated with plaques at different stages of development [37] were not considered in this study.

Recent studies have explored distinct molecular subgroups within psoriasis and the heterogeneity of inflammatory and cytokine networks dissecting the psoriasis transcriptome [38–40]. Our study added to those explorations, but took a unique approach to integrating histologically-measured clinical phenotypes with molecular phenotypes. Rather than dissecting the psoriasis transcriptome to identify subtypes with distinct molecular signatures, we first utilized histologic measurements of epidermal thickness to subtype psoriasis, and then identified different expression levels of the common psoriasis transcriptome in skin and different levels of circulating cytokines in blood between subtypes.

## Conclusions

All together, we applied histological stratification of thick and thin plaque psoriasis to identify molecular phenotypes with different expression levels of the psoriasis transcriptome. Different expression levels of the psoriasis transcriptome correlated with the amount of inflammatory cytokines in psoriasis skin and circulating blood. Overall, we were able to integrate histological stratification with molecular phenotyping as a way of exploring clinical phenotypes with different expression levels of psoriasis transcriptome and circulating cytokines.

## Supporting Information

S1 Fig Forward stepwise linear regression model to predict epidermal thickness. (PDF)

S1 Table Histological measures of epidermal thickness in a cohort of 609 patients. (PDF)
