# Supplementary material for: Histological Stratification of Thick and Thin Plaque Psoriasis Explores Molecular Phenotypes with Clinical Implications
Source: PLoS One. 2015 Jul 15;10(7):e0132454. doi: 10.1371/journal.pone.0132454 (PMC4503455; doi:10.1371/journal.pone.0132454)
Supplement: S1 Fig — (PDF) [file pone.0132454.s001.pdf]

SUPPLEMENTARY FIGURES

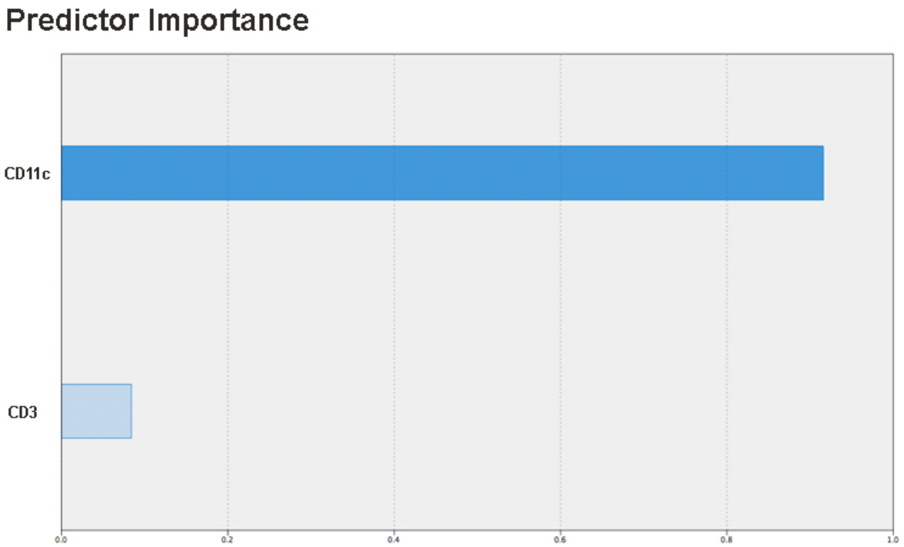

Effects

Source	Sum of Squares	df	Mean Square	F	Sig.	Importance
Corrected Model ▼	2,218,978.948	2	1,109,489.474	100.911	.000	
CD11C_DER_transformed	645,558.754	1	645,558.754	58.715	.000	0.916
CD3_DER_transformed	59,233.222	1	59,233.222	5.387	.022	0.084
Residual	1,396,329.849	127	10,994.723			
Corrected Total	3,615,308.796	129				

Coefficients

Model Term	Coefficient ▼	Std.Error	t	Sig.	95% Confidence Interval		Importance
					Lower	Upper	
Intercept	68.388	14.143	4.836	.000	40.402	96.374	
CD11C_DER_transformed	0.836	0.109	7.663	.000	0.620	1.052	0.916
CD3_DER_transformed	0.252	0.109	2.321	.022	0.037	0.468	0.084

S1 Fig. Forward stepwise linear regression model to predict epidermal thickness. To confirm the validity of clinical stratification by epidermal thickness, numbers of $CD3^+$ cells, $CD11c^+$ cells, DC-$LAMP^+$ cells, and $BDCA^+$ cells in the dermis are entered as determining pathogenic factors of psoriasis. Among 4 explanatory variables, number of $CD11c^+$ dendritic cells and $CD3^+$ T cells correlated with epidermal thickness (correlation coefficients; $CD11c^+$ cells = 0.836, $CD3^+$ cells = 0.252, p <0.05).
