# Supplementary material for: Histological Stratification of Thick and Thin Plaque Psoriasis Explores Molecular Phenotypes with Clinical Implications
Source: PLoS One. 2015 Jul 15;10(7):e0132454. doi: 10.1371/journal.pone.0132454 (PMC4503455; doi:10.1371/journal.pone.0132454)
Supplement: S1 Table — (PDF) [file pone.0132454.s002.pdf]

SUPPLEMENTARY TABLE

S1 Table. Histological measures of epidermal thickness in a cohort of 609 patients.

Patient	Epidermal thickness (µm)	
	Non-lesional skin	Lesional skin
1	108.9	490.4
2	87.9	314.8
3	118.1	416.8
4	132.2	343.9
5	101.9	202.1
6	144.5	434.9
7	95.6	342.4
8	97.8	357.5
9	102.1	404.2
10	139.9	404.8
11	57.8	111.5
12	95.3	423.4
13	102.1	484
14	76.3	224.5
15	95	425.7
16	77.6	285.8
17	88.9	546.5
18	100.7	472.3
19	157.3	493.8
20	119.2	545.8
21	93.7	605
22	66.1	406.1
23	102	461.3
24	132.1	732.1
25	129.2	485
26	91.1	472.1
27	163.9	262.3
28	118.7	399
29	101.7	361.9
30	104.8	532.2
31	46.6	243.2
32	55.5	215.6
33	43.2	152.1
34	75.2	260
35	55.9	399.4
36	57.5	337.7
37	65.4	247.3
38	75.4	327
39	56.7	380.6
40	37.5	339.3

S1 Table. Continued.

41	69.3	206.8
42	54.2	214.9
43	66.3	334.8
44	93	259.1
45	88.9	414.5
46	84.6	361.3
47	70.4	410.5
48	59.5	241.4
49	58.1	381.1
50	99.3	465.3
51	103.8	288.5
52	90.8	252.1
53	69.4	627.5
54	126.9	478.7
55	77.4	691.4
56	57	175.4
57	77.7	322.3
58	71.3	504.5
59	94.7	485
60	118.7	541.4
61	57	341.3
62	116.4	365.2
63	112.7	280.7
64	68.5	530.8
65	97.8	421
66	101.6	319.6
67	120.7	526.3
68	85.7	476.9
69	74.4	415.9
70	68.2	303.5
71	75	286.9
72	161.5	298.7
73	115.3	505.1
74	93.2	209.1
75	59.6	341.2
76	69.8	254.6
77	82.6	235.2
78	128.5	446
79	73.2	500.7
80	95.3	631.2
81	106.6	250.6
82	78.5	338.7

S1 Table. Continued.

83	60.1	237.1
84	74.8	281.7
85	58.2	262.3
86	74.2	173
87	96.9	488.4
88	72.4	417.9
89	110.3	182.1
90	95.8	615.3
91	82	332.2
92	85.7	235.7
93	103	490.1
94	84.8	454.4
95	70.1	547
96	121.2	410.3
97	97.9	357.5
98	97.1	348.2
99	90.1	271.8
100	96.2	748.2
101	97.8	332.1
102	113.8	512.5
103	143.9	339.4
104	134.1	595.6
105	108.9	391.9
106	38.8	305.2
107	61.9	196.5
108	38	215.4
109	72.3	168.3
110	74.6	400.6
111	78.4	418.5
112	71.8	344
113	81.3	320.2
114	67.2	148.8
115	78.3	450.7
116	74.3	342.9
117	71.6	189.4
118	87.3	379.1
119	45	273.4
120	88.1	223.9
121	94.3	268.1
122	62.3	318.9
123	58.3	415.5
124	102.3	558.3

S1 Table. Continued.

125	105	557.2
126	79.1	291
127	82.2	150.9
128	62.7	303.8
129	97.3	267.5
130	86.2	341.2
131	65.2	379.1
132	77.1	373.2
133	71.7	337.8
134	98.8	311.3
135	56.6	341.6
136	57.8	331.9
137	70.6	404
138	72.8	354.3
139	79.8	305.9
140	69.3	287.1
141	75.9	300.2
142	60	405.5
143	82.7	262.7
144	126.8	746.7
145	81.7	261.1
146	92.3	460.3
147	79	220.6
148	139.8	554.4
149	110.9	275.8
150	92.8	333
151	115.5	320.4
152	69.3	205.8
153	84.1	444.2
154	87.1	335.3
155	146.6	358.3
156	101.6	195.8
157	98.6	399.3
158	79.1	379.4
159	75.8	153.4
160	102.6	513.1
161	84.5	215.4
162	95.3	348.2
163	97.4	279
164	99.5	254.6
165	91.9	273.7
166	73.9	254.6

S1 Table. Continued.

167	124.1	279.2
168	119.9	223.3
169	125.1	412.2
170	110.1	555.6
171	63	458
172	79.1	355.3
173	78.7	205.1
174	99.6	311.5
175	88.3	416.5
176	74.9	146.2
177	133.6	402.3
178	78.7	238.9
179	120.2	515.3
180	69.7	385
181	80.1	572.8
182	57.8	372
183	89	522.4
184	95.6	563.7
185	93.1	765.1
186	113.8	402.3
187	78.2	214.3
188	83.6	157.2
189	96.8	260.9
190	85.1	258.4
191	79.4	304.9
192	78.5	195.1
193	73	238.6
194	79.1	340.5
195	77.6	251.7
196	67.6	421.8
197	68	175.2
198	74.8	182.7
199	63.7	573.8
200	39.6	318
201	76.1	377
202	83.4	346.2
203	113.9	602.9
204	63.5	318.5
205	90.5	373.1
206	69.7	218.2
207	81.1	386.2
208	79.3	418.4

S1 Table. Continued.

209	86	189.4
210	54.4	308.6
211	76.3	310.4
212	74.1	144.9
213	99.6	555.7
214	85	503.6
215	69.1	198.3
216	80.7	294.4
217	113.2	633.4
218	85.5	186.8
219	92	398.4
220	63.2	532.6
221	88.1	281.3
222	121.6	418.8
223	53.1	399.1
224	75	233.4
225	92.5	269.3
226	113.3	698.5
227	108.2	153.9
228	69.8	235.3
229	75.9	416.7
230	77.3	297.3
231	69.7	201.3
232	66.6	241.3
233	45.5	253.3
234	128.6	381.9
235	107	502.9
236	109.5	347.6
237	132.4	309.3
238	104.5	472.7
239	96.1	388
240	103.1	323.8
241	157.5	291.9
242	167.8	622.1
243	92.3	228
244	174.5	345.2
245	89.2	248.5
246	112.5	441.5
247	163.3	561.5
248	133.4	189.5
249	74.9	189.2
250	92.1	611.5

S1 Table. Continued.

251	89.2	411.7
252	137.8	267
253	100.3	531.8
254	155.6	204.2
255	184.8	457.8
256	96.9	206.2
257	64.1	196.2
258	68.3	265.5
259	103.9	586.2
260	100.8	402.3
261	102.4	180.4
262	79.6	192.3
263	87	268.9
264	84.4	267.8
265	92.3	230.3
266	156.2	503
267	94.9	274.7
268	95.8	351.6
269	133.5	535.2
270	134.1	363.9
271	62.7	295.9
272	111.2	202.7
273	112.3	513
274	107.4	497.5
275	146	386.8
276	99.7	407
277	81.5	367.9
278	118	245.1
279	76	190.3
280	62.4	201.6
281	104.4	361.6
282	68.1	229.5
283	133.1	299.9
284	95.1	408.8
285	75.9	457.9
286	77.3	446.1
287	89.7	391
288	83.8	395.6
289	103	529.7
290	116.2	608.7
291	72.6	483
292	87.9	332.7

S1 Table. Continued.

293	58.6	409.1
294	84.3	410.4
295	81.8	496.6
296	103.9	320.6
297	124.5	533.5
298	72.3	223.8
299	106.6	603.1
300	98.1	381.7
301	95.3	345.3
302	58.1	437
303	97.7	250.3
304	84.5	311.9
305	90.2	365.7
306	90.7	407.6
307	114.7	434.8
308	95.7	264.9
309	76.2	392.8
310	87.9	335.5
311	111.1	306.8
312	79.2	571.2
313	118.9	271.4
314	118.9	450.4
315	105	413.7
316	87.8	365.6
317	90.1	245.5
318	153.6	342.3
319	137.7	295.2
320	111.7	623.1
321	95.2	480.3
322	82.2	361.9
323	93.8	354.1
324	135.6	357.3
325	145.4	321.1
326	188.6	330
327	91.2	417.8
328	70.6	317.8
329	146.7	421.8
330	101.4	349.6
331	108.4	294.8
332	126.6	528.9
333	78.6	358.2
334	98.1	378.6

S1 Table. Continued.

335	93	388.5
336	125.5	178.2
337	69.8	398.6
338	94.3	425.6
339	59.6	225.1
340	57.4	167.8
341	69	292.3
342	106.3	294.1
343	63.1	142.4
344	99	489.5
345	77.2	417.1
346	80.5	316.2
347	54.7	265.1
348	88.4	244.2
349	81.9	325.7
350	72	391.3
351	161	543.2
352	110.7	258.8
353	82.9	485.1
354	97.5	510.3
355	106.7	354.7
356	93.3	280.2
357	140.8	257
358	79.3	192.7
359	106.6	388.4
360	97.3	338.6
361	75.3	302.5
362	152	389.1
363	83.5	470.1
364	84.3	287.3
365	103.8	453.4
366	88.6	368.6
367	52.4	386.2
368	89.9	562
369	65.3	146.2
370	52.4	377
371	84.6	617.6
372	50.9	416.3
373	79.3	540.7
374	71.5	277.2
375	85.7	168.4
376	83.9	656.6

S1 Table. Continued.

377	63.8	505.8
378	58	205.5
379	88	344.4
380	116.7	243.9
381	69.7	338.2
382	70.9	637.6
383	90.1	370.2
384	107.8	262.4
385	126.4	351.7
386	95.3	336.4
387	125.6	430.7
388	79	189.7
389	96.2	169.8
390	79.2	457.9
391	62.4	537.6
392	95.4	237.5
393	70.4	450.8
394	75	237
395	69.7	418.3
396	89.5	453.6
397	119.2	688.8
398	53.1	424.3
399	81.5	343.5
400	86.8	501.9
401	115.6	523.8
402	71.6	332.1
403	73.6	258
404	86	365.8
405	74.5	152.6
406	57.2	224.4
407	81.6	399
408	74.9	399.5
409	68.7	447.4
410	55.7	404.2
411	66.5	232
412	93.2	431.7
413	81.9	507.2
414	88.6	403.8
415	63.5	228.6
416	82.4	595.5
417	78.6	541.9
418	87.8	572.9

S1 Table. Continued.

419	104.7	509.3
420	75.9	507
421	73.3	453.3
422	64.6	284.5
423	101.3	434.6
424	81.6	250.1
425	73.9	247.9
426	76.9	389.2
427	136.5	322.9
428	48.1	325.8
429	86.7	420.5
430	80.9	308.1
431	78.6	553.9
432	106.2	584.8
433	79.2	378.8
434	74.4	273.6
435	116.2	472.5
436	95	557.3
437	91.7	393.8
438	92.9	279.5
439	65.2	288
440	108.7	258.3
441	132.3	452.3
442	71.5	176.7
443	57.4	269.2
444	78.1	214.6
445	83.5	243.2
446	88.8	511.7
447	49.5	199.3
448	77.3	343.8
449	118.4	299.3
450	67.9	233.8
451	129.6	557.4
452	74	472.5
453	71.1	218.3
454	182.1	373.5
455	148	406.7
456	135.9	322.2
457	70.6	304.4
458	116.2	472.6
459	86.3	335
460	69.9	300.5

S1 Table. Continued.

461	80.9	310.4
462	67.8	270
463	126.9	508
464	92.4	450.9
465	136	336.2
466	70.4	294.7
467	149	232.8
468	96.3	391.7
469	101.1	465.9
470	135.8	410.6
471	103.3	301.2
472	61.9	238.1
473	82.2	270.3
474	84.6	299.1
475	69.8	240.8
476	93.5	192.6
477	100.2	483.5
478	91.8	283.3
479	84.5	303.9
480	55.8	306.7
481	66.8	196.3
482	97.3	457.1
483	105.7	441.4
484	107.7	336.8
485	89.4	164
486	87.5	210
487	102.8	159.9
488	96.3	491.9
489	100	399.2
490	126.2	196.3
491	101.7	505.3
492	64.9	231.5
493	77.4	373
494	68.4	225.4
495	78.6	355.1
496	80.4	237.1
497	66.6	320.2
498	65.3	217.1
499	43.4	316.6
500	116.1	367.7
501	72.2	400
502	89.1	241.7

S1 Table. Continued.

503	72.1	357.9
504	52.9	455.5
505	64.7	565.5
506	64.4	294.3
507	55.9	599.5
508	72.5	317.3
509	131.1	242.9
510	70.9	556.5
511	57.6	153.7
512	120.5	234.1
513	136	379.2
514	94.4	238.5
515	78.4	453.3
516	52.4	220.2
517	66	491.5
518	78	304.4
519	73.5	469
520	71.1	445.4
521	58.7	115.7
522	111.5	413.2
523	63.2	309.7
524	74.3	513.3
525	103.2	643.3
526	95.3	268.4
527	107.4	308.9
528	109.1	143.3
529	91.8	535.7
530	84	653.9
531	81.1	278.8
532	110.6	158.8
533	62.6	275.7
534	88.8	336.1
535	73.7	357.1
536	72.8	179
537	54.2	381.6
538	76.5	375
539	100.8	452.6
540	172.8	364.4
541	126.8	383.4
542	109.3	574.5
543	129.2	353.1
544	68.5	280.1

S1 Table. Continued.

545	63.7	379.1
546	75.7	335
547	87.3	314.2
548	109.2	371.9
549	79.7	349.5
550	56.9	103.5
551	95.7	453.1
552	89.2	178.2
553	62.4	481.6
554	107.1	175.3
555	94.7	296
556	76.7	404.2
557	107.1	441.7
558	94.9	140
559	82.1	242.3
560	71.5	384.9
561	97.2	174.7
562	89.8	241.1
563	95.1	359.2
564	96	422.2
565	112.6	326.8
566	118.5	343.3
567	91.8	309.3
568	77.6	356.6
569	57.1	260.1
570	88.4	547.9
571	77.5	314.5
572	75	554.5
573	102.6	216.5
574	87.1	385.5
575	107.7	366.3
576	90.9	403.8
577	162.1	268.3
578	64.9	395.9
579	75.3	502.9
580	98.9	448
581	109.1	491.3
582	64.8	483.7
583	72.8	369.1
584	75.6	402
585	81.1	399.1
586	73.8	415.6

S1 Table. Continued.

587	66.5	97.5
588	64.6	161.7
589	83.9	423.7
590	69	319.6
591	66.8	283.5
592	78.2	371.8
593	96.1	266.4
594	58.5	328.8
595	71.5	136
596	86.2	281.4
597	70.9	347.4
598	66.4	409.8
599	52.3	294.4
600	103.3	609.7
601	71.9	418.4
602	98.7	274.6
603	114	232.8
604	82	342.8
605	79.6	271.9
606	77.7	270.2
607	92.6	328.6
608	103.5	254.3
609	73.3	844.8
